# Supplementary material for: Modeling glioblastoma heterogeneity as a dynamic network of cell states
Source: Mol Syst Biol. 2021 Sep 16;17(9):e10105. doi: 10.15252/msb.202010105 (PMC8444284; doi:10.15252/msb.202010105)
Supplement: Supplementary file 5 — Source Data for Figure 3 [file MSB-17-e10105-s001.zip › Figure3A_sourcedata/GSEA_3065/nosigdb_state1.GseaPreranked.1622718409535/RICHARDS_NOWAKOWSKI_SCIENCE_2017_OPC_UPREG.html]

Details for gene set RICHARDS\_NOWAKOWSKI\_SCIENCE\_2017\_OPC\_UPREG[GSEA]

|  || Dataset | state1 |
| Phenotype | NoPhenotypeAvailable |
| Upregulated in class | na\_neg |
| GeneSet | RICHARDS\_NOWAKOWSKI\_SCIENCE\_2017\_OPC\_UPREG |
| Enrichment Score (ES) | -0.6917307 |
| Normalized Enrichment Score (NES) | -2.8109875 |
| Nominal p-value | 0.0 |
| FDR q-value | 0.0 |
| FWER p-Value | 0.0 |
Table: GSEA Results Summary

  

Fig 1: Enrichment plot: RICHARDS\_NOWAKOWSKI\_SCIENCE\_2017\_OPC\_UPREG      
 Profile of the Running ES Score & Positions of GeneSet Members on the Rank Ordered List

  

| PROBE | GENE SYMBOL | GENE\_TITLE | RANK IN GENE LIST | RANK METRIC SCORE | RUNNING ES | CORE ENRICHMENT || 1 | CCND1 |  |  | 29 | 0.566 | 0.0055 | No |
| 2 | TMEFF2 |  |  | 36 | 0.526 | 0.0129 | No |
| 3 | DSTN |  |  | 70 | 0.417 | 0.0157 | No |
| 4 | PHLDA1 |  |  | 83 | 0.389 | 0.0204 | No |
| 5 | DBI |  |  | 110 | 0.352 | 0.0230 | No |
| 6 | KHDRBS3 |  |  | 113 | 0.349 | 0.0281 | No |
| 7 | OCIAD2 |  |  | 135 | 0.332 | 0.0309 | No |
| 8 | ANKS1B |  |  | 193 | 0.298 | 0.0294 | No |
| 9 | SLC25A5 |  |  | 254 | 0.276 | 0.0273 | No |
| 10 | NME1 |  |  | 395 | 0.227 | 0.0161 | No |
| 11 | NT5E |  |  | 462 | 0.213 | 0.0125 | No |
| 12 | ARL4A |  |  | 497 | 0.206 | 0.0120 | No |
| 13 | LYPD1 |  |  | 508 | 0.205 | 0.0141 | No |
| 14 | GOLIM4 |  |  | 525 | 0.200 | 0.0154 | No |
| 15 | PDE1C |  |  | 609 | 0.184 | 0.0095 | No |
| 16 | SHISA9 |  |  | 741 | 0.166 | -0.0017 | No |
| 17 | CISD1 |  |  | 757 | 0.165 | -0.0007 | No |
| 18 | NDUFB3 |  |  | 766 | 0.162 | 0.0009 | No |
| 19 | CNTN1 |  |  | 911 | 0.143 | -0.0120 | No |
| 20 | DENR |  |  | 948 | 0.140 | -0.0136 | No |
| 21 | ACP1 |  |  | 1000 | 0.134 | -0.0170 | No |
| 22 | NTM |  |  | 1002 | 0.134 | -0.0150 | No |
| 23 | PRDX3 |  |  | 1003 | 0.133 | -0.0130 | No |
| 24 | SPRY2 |  |  | 1171 | 0.115 | -0.0288 | No |
| 25 | TNFRSF21 |  |  | 1222 | 0.110 | -0.0323 | No |
| 26 | SULF2 |  |  | 1391 | 0.096 | -0.0484 | No |
| 27 | DUSP6 |  |  | 1401 | 0.095 | -0.0479 | No |
| 28 | ATP6V0E1 |  |  | 1421 | 0.094 | -0.0485 | No |
| 29 | RIN2 |  |  | 1450 | 0.092 | -0.0500 | No |
| 30 | FABP5 |  |  | 1470 | 0.091 | -0.0507 | No |
| 31 | PDLIM5 |  |  | 1528 | 0.087 | -0.0553 | No |
| 32 | NRIP3 |  |  | 1645 | 0.079 | -0.0662 | No |
| 33 | WDR83OS |  |  | 1667 | 0.077 | -0.0673 | No |
| 34 | CA10 |  |  | 1674 | 0.077 | -0.0667 | No |
| 35 | LIMA1 |  |  | 1678 | 0.077 | -0.0659 | No |
| 36 | SNX7 |  |  | 1724 | 0.074 | -0.0695 | No |
| 37 | SLC1A1 |  |  | 1828 | 0.068 | -0.0792 | No |
| 38 | FGF12 |  |  | 1872 | 0.066 | -0.0827 | No |
| 39 | KIF13A |  |  | 1893 | 0.065 | -0.0838 | No |
| 40 | RSU1 |  |  | 1904 | 0.065 | -0.0839 | No |
| 41 | TNS3 |  |  | 1918 | 0.064 | -0.0843 | No |
| 42 | GLTP |  |  | 1951 | 0.063 | -0.0867 | No |
| 43 | MAMLD1 |  |  | 2206 | 0.051 | -0.1125 | No |
| 44 | CUTC |  |  | 2258 | 0.049 | -0.1171 | No |
| 45 | FRMD6 |  |  | 2464 | 0.041 | -0.1379 | No |
| 46 | NDUFAF2 |  |  | 2587 | 0.037 | -0.1501 | No |
| 47 | RPS27L |  |  | 2723 | 0.032 | -0.1638 | No |
| 48 | CFLAR |  |  | 2958 | 0.026 | -0.1878 | No |
| 49 | SFT2D1 |  |  | 2961 | 0.026 | -0.1877 | No |
| 50 | STARD7 |  |  | 3048 | 0.024 | -0.1963 | No |
| 51 | GNG12 |  |  | 3088 | 0.023 | -0.2000 | No |
| 52 | LNX1 |  |  | 3100 | 0.022 | -0.2009 | No |
| 53 | GUCD1 |  |  | 3154 | 0.021 | -0.2061 | No |
| 54 | CHIC2 |  |  | 3170 | 0.021 | -0.2073 | No |
| 55 | RAP2C |  |  | 3187 | 0.021 | -0.2087 | No |
| 56 | CHRM3 |  |  | 3269 | 0.019 | -0.2169 | No |
| 57 | DST |  |  | 3373 | 0.017 | -0.2274 | No |
| 58 | CDCA7L |  |  | 3390 | 0.016 | -0.2288 | No |
| 59 | BCAT1 |  |  | 3755 | 0.009 | -0.2668 | No |
| 60 | ETV5 |  |  | 3894 | 0.006 | -0.2811 | No |
| 61 | ACTN4 |  |  | 3909 | 0.006 | -0.2825 | No |
| 62 | WASF1 |  |  | 3937 | 0.006 | -0.2853 | No |
| 63 | PTPRE |  |  | 4002 | 0.004 | -0.2919 | No |
| 64 | LITAF |  |  | 4039 | 0.003 | -0.2956 | No |
| 65 | UGDH |  |  | 4106 | 0.002 | -0.3025 | No |
| 66 | STXBP3 |  |  | 4241 | -0.000 | -0.3165 | No |
| 67 | FOXN2 |  |  | 4398 | -0.003 | -0.3328 | No |
| 68 | SASH1 |  |  | 4498 | -0.004 | -0.3431 | No |
| 69 | TAF2 |  |  | 4536 | -0.005 | -0.3469 | No |
| 70 | GLDC |  |  | 4543 | -0.005 | -0.3474 | No |
| 71 | STARD4 |  |  | 4548 | -0.005 | -0.3477 | No |
| 72 | BMP7 |  |  | 4562 | -0.006 | -0.3490 | No |
| 73 | TCF7L2 |  |  | 4707 | -0.008 | -0.3640 | No |
| 74 | LDHB |  |  | 4712 | -0.008 | -0.3643 | No |
| 75 | ABI1 |  |  | 4714 | -0.008 | -0.3643 | No |
| 76 | CLDND1 |  |  | 4823 | -0.009 | -0.3754 | No |
| 77 | UBE3B |  |  | 4913 | -0.011 | -0.3846 | No |
| 78 | CAT |  |  | 4951 | -0.012 | -0.3883 | No |
| 79 | PKP4 |  |  | 5005 | -0.013 | -0.3936 | No |
| 80 | REEP3 |  |  | 5071 | -0.014 | -0.4002 | No |
| 81 | MTPAP |  |  | 5418 | -0.019 | -0.4361 | No |
| 82 | MED11 |  |  | 5426 | -0.019 | -0.4366 | No |
| 83 | MYBL1 |  |  | 5455 | -0.019 | -0.4392 | No |
| 84 | RABGAP1L |  |  | 5486 | -0.020 | -0.4421 | No |
| 85 | STRN |  |  | 5548 | -0.021 | -0.4481 | No |
| 86 | COPB2 |  |  | 5695 | -0.023 | -0.4631 | No |
| 87 | NUP50 |  |  | 5803 | -0.025 | -0.4739 | No |
| 88 | DHCR24 |  |  | 5843 | -0.026 | -0.4775 | No |
| 89 | RAB9A |  |  | 5858 | -0.026 | -0.4786 | No |
| 90 | PGM3 |  |  | 5937 | -0.028 | -0.4863 | No |
| 91 | BPNT1 |  |  | 5979 | -0.029 | -0.4902 | No |
| 92 | PCBP4 |  |  | 6003 | -0.029 | -0.4922 | No |
| 93 | UIMC1 |  |  | 6059 | -0.030 | -0.4975 | No |
| 94 | SATB1 |  |  | 6070 | -0.030 | -0.4981 | No |
| 95 | B9D1 |  |  | 6089 | -0.031 | -0.4995 | No |
| 96 | CALCRL |  |  | 6193 | -0.033 | -0.5098 | No |
| 97 | KHSRP |  |  | 6323 | -0.035 | -0.5227 | No |
| 98 | RAP2A |  |  | 6372 | -0.036 | -0.5272 | No |
| 99 | NOMO1 |  |  | 6404 | -0.037 | -0.5299 | No |
| 100 | AFAP1L2 |  |  | 6415 | -0.037 | -0.5304 | No |
| 101 | CPEB2 |  |  | 6458 | -0.038 | -0.5342 | No |
| 102 | SPRY4 |  |  | 6504 | -0.039 | -0.5383 | No |
| 103 | C1orf21 |  |  | 6720 | -0.043 | -0.5602 | No |
| 104 | NOC3L |  |  | 6757 | -0.044 | -0.5633 | No |
| 105 | MAP4K5 |  |  | 6807 | -0.045 | -0.5677 | No |
| 106 | ZNF516 |  |  | 6867 | -0.046 | -0.5732 | No |
| 107 | DDAH1 |  |  | 6943 | -0.049 | -0.5803 | No |
| 108 | FARP2 |  |  | 7039 | -0.051 | -0.5895 | No |
| 109 | E2F3 |  |  | 7064 | -0.052 | -0.5912 | No |
| 110 | ZC3HAV1 |  |  | 7178 | -0.055 | -0.6022 | No |
| 111 | RAB31 |  |  | 7256 | -0.057 | -0.6094 | No |
| 112 | CNP |  |  | 7303 | -0.058 | -0.6133 | No |
| 113 | IQSEC1 |  |  | 7311 | -0.058 | -0.6132 | No |
| 114 | ALDH6A1 |  |  | 7321 | -0.059 | -0.6132 | No |
| 115 | FNTA |  |  | 7409 | -0.061 | -0.6214 | No |
| 116 | DOCK1 |  |  | 7505 | -0.064 | -0.6304 | No |
| 117 | RALGPS2 |  |  | 7602 | -0.066 | -0.6394 | No |
| 118 | ARHGAP42 |  |  | 7652 | -0.068 | -0.6435 | No |
| 119 | GRIA4 |  |  | 7708 | -0.070 | -0.6482 | No |
| 120 | PCDH17 |  |  | 7749 | -0.072 | -0.6513 | No |
| 121 | TP53INP1 |  |  | 7767 | -0.072 | -0.6520 | No |
| 122 | RNF144A |  |  | 7775 | -0.072 | -0.6517 | No |
| 123 | TNRC6C |  |  | 7785 | -0.073 | -0.6515 | No |
| 124 | KLHL42 |  |  | 7800 | -0.073 | -0.6518 | No |
| 125 | DNER |  |  | 7852 | -0.075 | -0.6560 | No |
| 126 | NXPH1 |  |  | 7920 | -0.078 | -0.6619 | No |
| 127 | MRPL32 |  |  | 7924 | -0.078 | -0.6610 | No |
| 128 | SEPT8 |  |  | 7971 | -0.080 | -0.6646 | No |
| 129 | SMAD5 |  |  | 8035 | -0.083 | -0.6699 | No |
| 130 | CAMSAP2 |  |  | 8070 | -0.084 | -0.6722 | No |
| 131 | DSCAM |  |  | 8074 | -0.084 | -0.6713 | No |
| 132 | EVI5 |  |  | 8095 | -0.085 | -0.6721 | No |
| 133 | PPIB |  |  | 8099 | -0.085 | -0.6711 | No |
| 134 | SKIL |  |  | 8133 | -0.087 | -0.6732 | No |
| 135 | SSR1 |  |  | 8183 | -0.089 | -0.6770 | No |
| 136 | SH3D19 |  |  | 8185 | -0.089 | -0.6758 | No |
| 137 | SAMD8 |  |  | 8220 | -0.090 | -0.6780 | No |
| 138 | CHST3 |  |  | 8240 | -0.091 | -0.6786 | No |
| 139 | LRRK2 |  |  | 8253 | -0.092 | -0.6784 | No |
| 140 | LPP |  |  | 8255 | -0.092 | -0.6772 | No |
| 141 | PPP2R2B |  |  | 8319 | -0.095 | -0.6823 | No |
| 142 | TCF7L1 |  |  | 8342 | -0.096 | -0.6831 | No |
| 143 | DENND5A |  |  | 8396 | -0.099 | -0.6872 | No |
| 144 | EPB41L2 |  |  | 8402 | -0.099 | -0.6862 | No |
| 145 | KIAA0232 |  |  | 8412 | -0.100 | -0.6857 | No |
| 146 | KLHL5 |  |  | 8438 | -0.101 | -0.6867 | No |
| 147 | LCORL |  |  | 8458 | -0.102 | -0.6872 | No |
| 148 | GRB14 |  |  | 8474 | -0.103 | -0.6872 | No |
| 149 | GNB4 |  |  | 8485 | -0.104 | -0.6866 | No |
| 150 | TMX1 |  |  | 8524 | -0.107 | -0.6890 | No |
| 151 | ARNT2 |  |  | 8528 | -0.107 | -0.6877 | No |
| 152 | CASK |  |  | 8532 | -0.107 | -0.6864 | No |
| 153 | UBL3 |  |  | 8573 | -0.110 | -0.6889 | No |
| 154 | FBXO7 |  |  | 8575 | -0.110 | -0.6874 | No |
| 155 | PEAK1 |  |  | 8607 | -0.112 | -0.6889 | No |
| 156 | TENM1 |  |  | 8617 | -0.112 | -0.6881 | No |
| 157 | UNC80 |  |  | 8620 | -0.113 | -0.6866 | No |
| 158 | COL11A1 |  |  | 8666 | -0.115 | -0.6896 | No |
| 159 | TMEM206 |  |  | 8669 | -0.115 | -0.6881 | No |
| 160 | DNM3 |  |  | 8701 | -0.117 | -0.6895 | No |
| 161 | BLM |  |  | 8723 | -0.119 | -0.6899 | Yes |
| 162 | SERPINE2 |  |  | 8736 | -0.120 | -0.6894 | Yes |
| 163 | MAP3K1 |  |  | 8750 | -0.121 | -0.6889 | Yes |
| 164 | KAT2B |  |  | 8754 | -0.122 | -0.6873 | Yes |
| 165 | NAPEPLD |  |  | 8761 | -0.122 | -0.6861 | Yes |
| 166 | SAR1B |  |  | 8778 | -0.123 | -0.6859 | Yes |
| 167 | GCC2 |  |  | 8799 | -0.124 | -0.6861 | Yes |
| 168 | PCMTD2 |  |  | 8830 | -0.127 | -0.6874 | Yes |
| 169 | TRIB2 |  |  | 8853 | -0.129 | -0.6877 | Yes |
| 170 | ZCCHC24 |  |  | 8878 | -0.132 | -0.6882 | Yes |
| 171 | CSGALNACT1 |  |  | 8884 | -0.132 | -0.6867 | Yes |
| 172 | SRPRB |  |  | 8910 | -0.135 | -0.6873 | Yes |
| 173 | NTN1 |  |  | 8923 | -0.136 | -0.6865 | Yes |
| 174 | NTRK3 |  |  | 8930 | -0.137 | -0.6851 | Yes |
| 175 | PHYHIPL |  |  | 8937 | -0.137 | -0.6836 | Yes |
| 176 | SLC2A13 |  |  | 8942 | -0.138 | -0.6819 | Yes |
| 177 | MT3 |  |  | 8954 | -0.139 | -0.6810 | Yes |
| 178 | UFL1 |  |  | 8970 | -0.140 | -0.6804 | Yes |
| 179 | SHC3 |  |  | 8972 | -0.141 | -0.6784 | Yes |
| 180 | C2orf80 |  |  | 8993 | -0.142 | -0.6784 | Yes |
| 181 | LEPROT |  |  | 8994 | -0.142 | -0.6762 | Yes |
| 182 | CTTNBP2 |  |  | 9021 | -0.144 | -0.6767 | Yes |
| 183 | FIBIN |  |  | 9036 | -0.145 | -0.6760 | Yes |
| 184 | GATM |  |  | 9049 | -0.147 | -0.6750 | Yes |
| 185 | KCND3 |  |  | 9051 | -0.147 | -0.6729 | Yes |
| 186 | LRRN1 |  |  | 9054 | -0.147 | -0.6709 | Yes |
| 187 | ST3GAL5 |  |  | 9085 | -0.151 | -0.6717 | Yes |
| 188 | ZADH2 |  |  | 9092 | -0.151 | -0.6701 | Yes |
| 189 | THY1 |  |  | 9119 | -0.153 | -0.6705 | Yes |
| 190 | ZNF37A |  |  | 9125 | -0.154 | -0.6687 | Yes |
| 191 | NLGN1 |  |  | 9131 | -0.155 | -0.6668 | Yes |
| 192 | CADM2 |  |  | 9141 | -0.157 | -0.6654 | Yes |
| 193 | VCAN |  |  | 9151 | -0.158 | -0.6639 | Yes |
| 194 | PLOD2 |  |  | 9169 | -0.161 | -0.6633 | Yes |
| 195 | LRP1B |  |  | 9174 | -0.162 | -0.6612 | Yes |
| 196 | RNF157 |  |  | 9191 | -0.164 | -0.6604 | Yes |
| 197 | ANGPTL2 |  |  | 9201 | -0.166 | -0.6589 | Yes |
| 198 | KANK1 |  |  | 9229 | -0.169 | -0.6591 | Yes |
| 199 | AASS |  |  | 9237 | -0.171 | -0.6573 | Yes |
| 200 | SEPT2 |  |  | 9246 | -0.172 | -0.6555 | Yes |
| 201 | EPN2 |  |  | 9249 | -0.172 | -0.6531 | Yes |
| 202 | ATCAY |  |  | 9276 | -0.176 | -0.6531 | Yes |
| 203 | IL1RAP |  |  | 9288 | -0.178 | -0.6516 | Yes |
| 204 | BAALC |  |  | 9292 | -0.179 | -0.6492 | Yes |
| 205 | ZMAT3 |  |  | 9300 | -0.181 | -0.6472 | Yes |
| 206 | TMX3 |  |  | 9328 | -0.186 | -0.6472 | Yes |
| 207 | TNK2 |  |  | 9333 | -0.187 | -0.6448 | Yes |
| 208 | PDGFA |  |  | 9341 | -0.188 | -0.6427 | Yes |
| 209 | MPZL1 |  |  | 9345 | -0.189 | -0.6401 | Yes |
| 210 | TAOK3 |  |  | 9351 | -0.190 | -0.6378 | Yes |
| 211 | SLC22A17 |  |  | 9357 | -0.191 | -0.6354 | Yes |
| 212 | HSP90B1 |  |  | 9365 | -0.193 | -0.6332 | Yes |
| 213 | NLGN4X |  |  | 9377 | -0.196 | -0.6314 | Yes |
| 214 | GPR155 |  |  | 9382 | -0.196 | -0.6289 | Yes |
| 215 | SIRT2 |  |  | 9386 | -0.198 | -0.6262 | Yes |
| 216 | HIP1 |  |  | 9394 | -0.200 | -0.6239 | Yes |
| 217 | RFTN2 |  |  | 9402 | -0.200 | -0.6216 | Yes |
| 218 | TM7SF3 |  |  | 9409 | -0.202 | -0.6191 | Yes |
| 219 | RDH11 |  |  | 9422 | -0.205 | -0.6173 | Yes |
| 220 | HSD17B12 |  |  | 9436 | -0.208 | -0.6155 | Yes |
| 221 | SEMA6D |  |  | 9442 | -0.210 | -0.6128 | Yes |
| 222 | APCDD1 |  |  | 9452 | -0.214 | -0.6105 | Yes |
| 223 | PHLPP1 |  |  | 9463 | -0.217 | -0.6083 | Yes |
| 224 | YIPF6 |  |  | 9473 | -0.220 | -0.6059 | Yes |
| 225 | PIK3R1 |  |  | 9474 | -0.220 | -0.6026 | Yes |
| 226 | ZNF462 |  |  | 9480 | -0.222 | -0.5998 | Yes |
| 227 | ITM2B |  |  | 9486 | -0.223 | -0.5969 | Yes |
| 228 | ZEB1 |  |  | 9492 | -0.224 | -0.5940 | Yes |
| 229 | SERINC5 |  |  | 9510 | -0.228 | -0.5924 | Yes |
| 230 | DAG1 |  |  | 9514 | -0.229 | -0.5892 | Yes |
| 231 | IL6ST |  |  | 9519 | -0.231 | -0.5861 | Yes |
| 232 | PDE4B |  |  | 9522 | -0.231 | -0.5829 | Yes |
| 233 | TRIO |  |  | 9524 | -0.231 | -0.5795 | Yes |
| 234 | HSPA5 |  |  | 9533 | -0.234 | -0.5768 | Yes |
| 235 | SPRY1 |  |  | 9540 | -0.236 | -0.5738 | Yes |
| 236 | NLGN3 |  |  | 9554 | -0.241 | -0.5715 | Yes |
| 237 | ITPR2 |  |  | 9560 | -0.244 | -0.5683 | Yes |
| 238 | PRKCA |  |  | 9569 | -0.248 | -0.5654 | Yes |
| 239 | FYN |  |  | 9575 | -0.250 | -0.5622 | Yes |
| 240 | MAGI1 |  |  | 9576 | -0.250 | -0.5584 | Yes |
| 241 | GLCCI1 |  |  | 9579 | -0.253 | -0.5548 | Yes |
| 242 | PCDHGC3 |  |  | 9588 | -0.255 | -0.5517 | Yes |
| 243 | TJP1 |  |  | 9592 | -0.257 | -0.5482 | Yes |
| 244 | DOCK10 |  |  | 9599 | -0.258 | -0.5449 | Yes |
| 245 | ERBB3 |  |  | 9601 | -0.258 | -0.5411 | Yes |
| 246 | DPP6 |  |  | 9606 | -0.262 | -0.5375 | Yes |
| 247 | SPCS3 |  |  | 9609 | -0.263 | -0.5338 | Yes |
| 248 | EMC10 |  |  | 9611 | -0.263 | -0.5299 | Yes |
| 249 | TMEM132B |  |  | 9613 | -0.264 | -0.5260 | Yes |
| 250 | SEZ6L |  |  | 9628 | -0.272 | -0.5233 | Yes |
| 251 | MAML2 |  |  | 9642 | -0.277 | -0.5205 | Yes |
| 252 | APBB2 |  |  | 9645 | -0.277 | -0.5165 | Yes |
| 253 | ABAT |  |  | 9646 | -0.278 | -0.5123 | Yes |
| 254 | ITGAV |  |  | 9650 | -0.282 | -0.5083 | Yes |
| 255 | SPATA6 |  |  | 9657 | -0.286 | -0.5046 | Yes |
| 256 | LRRN3 |  |  | 9659 | -0.286 | -0.5004 | Yes |
| 257 | HIPK2 |  |  | 9660 | -0.286 | -0.4961 | Yes |
| 258 | SETD5 |  |  | 9663 | -0.291 | -0.4919 | Yes |
| 259 | PLEKHH2 |  |  | 9666 | -0.291 | -0.4877 | Yes |
| 260 | PHLDB1 |  |  | 9671 | -0.294 | -0.4836 | Yes |
| 261 | SOX2 |  |  | 9680 | -0.298 | -0.4799 | Yes |
| 262 | SNX22 |  |  | 9681 | -0.299 | -0.4754 | Yes |
| 263 | LRRC4C |  |  | 9682 | -0.299 | -0.4709 | Yes |
| 264 | BCHE |  |  | 9684 | -0.300 | -0.4664 | Yes |
| 265 | NTRK2 |  |  | 9692 | -0.307 | -0.4625 | Yes |
| 266 | PDIA6 |  |  | 9701 | -0.314 | -0.4586 | Yes |
| 267 | PLP1 |  |  | 9702 | -0.314 | -0.4539 | Yes |
| 268 | ILDR2 |  |  | 9708 | -0.323 | -0.4495 | Yes |
| 269 | DSEL |  |  | 9714 | -0.327 | -0.4451 | Yes |
| 270 | SLC35F1 |  |  | 9715 | -0.327 | -0.4401 | Yes |
| 271 | CSPG5 |  |  | 9721 | -0.330 | -0.4356 | Yes |
| 272 | MMP16 |  |  | 9724 | -0.335 | -0.4308 | Yes |
| 273 | MYO10 |  |  | 9725 | -0.335 | -0.4257 | Yes |
| 274 | IDH1 |  |  | 9737 | -0.345 | -0.4216 | Yes |
| 275 | HAS2 |  |  | 9739 | -0.350 | -0.4164 | Yes |
| 276 | PCDH9 |  |  | 9746 | -0.353 | -0.4117 | Yes |
| 277 | P2RX7 |  |  | 9755 | -0.360 | -0.4071 | Yes |
| 278 | SDC3 |  |  | 9756 | -0.360 | -0.4016 | Yes |
| 279 | ITM2C |  |  | 9758 | -0.363 | -0.3962 | Yes |
| 280 | SCD |  |  | 9762 | -0.370 | -0.3910 | Yes |
| 281 | MARCKS |  |  | 9763 | -0.370 | -0.3853 | Yes |
| 282 | ETV1 |  |  | 9764 | -0.371 | -0.3797 | Yes |
| 283 | CHD7 |  |  | 9769 | -0.379 | -0.3744 | Yes |
| 284 | LRP1 |  |  | 9771 | -0.381 | -0.3688 | Yes |
| 285 | CADM4 |  |  | 9775 | -0.385 | -0.3632 | Yes |
| 286 | OLIG2 |  |  | 9776 | -0.386 | -0.3574 | Yes |
| 287 | SCARB2 |  |  | 9781 | -0.392 | -0.3519 | Yes |
| 288 | B2M |  |  | 9791 | -0.408 | -0.3466 | Yes |
| 289 | KIZ |  |  | 9793 | -0.416 | -0.3404 | Yes |
| 290 | ITGB8 |  |  | 9794 | -0.418 | -0.3341 | Yes |
| 291 | LAMA4 |  |  | 9800 | -0.425 | -0.3282 | Yes |
| 292 | QKI |  |  | 9803 | -0.430 | -0.3219 | Yes |
| 293 | CANX |  |  | 9804 | -0.431 | -0.3154 | Yes |
| 294 | PDGFRA |  |  | 9806 | -0.440 | -0.3088 | Yes |
| 295 | WSCD1 |  |  | 9808 | -0.448 | -0.3021 | Yes |
| 296 | ABHD2 |  |  | 9812 | -0.476 | -0.2952 | Yes |
| 297 | C1orf61 |  |  | 9813 | -0.479 | -0.2880 | Yes |
| 298 | LSAMP |  |  | 9814 | -0.481 | -0.2807 | Yes |
| 299 | ALCAM |  |  | 9818 | -0.493 | -0.2735 | Yes |
| 300 | GPM6A |  |  | 9820 | -0.499 | -0.2661 | Yes |
| 301 | SEPT7 |  |  | 9821 | -0.500 | -0.2585 | Yes |
| 302 | S100B |  |  | 9822 | -0.507 | -0.2508 | Yes |
| 303 | EDNRB |  |  | 9824 | -0.509 | -0.2432 | Yes |
| 304 | PGRMC1 |  |  | 9826 | -0.516 | -0.2355 | Yes |
| 305 | SLC44A1 |  |  | 9831 | -0.527 | -0.2279 | Yes |
| 306 | CD9 |  |  | 9832 | -0.544 | -0.2197 | Yes |
| 307 | PLAT |  |  | 9833 | -0.550 | -0.2114 | Yes |
| 308 | PON2 |  |  | 9835 | -0.554 | -0.2031 | Yes |
| 309 | SCD5 |  |  | 9838 | -0.576 | -0.1946 | Yes |
| 310 | LAPTM4B |  |  | 9840 | -0.579 | -0.1859 | Yes |
| 311 | OLIG1 |  |  | 9845 | -0.637 | -0.1767 | Yes |
| 312 | GRIA2 |  |  | 9846 | -0.638 | -0.1670 | Yes |
| 313 | FGFBP3 |  |  | 9850 | -0.651 | -0.1575 | Yes |
| 314 | MAP2 |  |  | 9857 | -0.735 | -0.1470 | Yes |
| 315 | NOVA1 |  |  | 9858 | -0.741 | -0.1357 | Yes |
| 316 | SEMA5A |  |  | 9860 | -0.749 | -0.1245 | Yes |
| 317 | KCNQ1OT1 |  |  | 9861 | -0.751 | -0.1131 | Yes |
| 318 | SOX6 |  |  | 9863 | -0.798 | -0.1011 | Yes |
| 319 | PTPRZ1 |  |  | 9869 | -0.918 | -0.0878 | Yes |
| 320 | PMP2 |  |  | 9871 | -0.982 | -0.0730 | Yes |
| 321 | GPM6B |  |  | 9873 | -1.091 | -0.0566 | Yes |
| 322 | PTN |  |  | 9874 | -1.107 | -0.0398 | Yes |
| 323 | BCAN |  |  | 9878 | -1.175 | -0.0223 | Yes |
| 324 | SCRG1 |  |  | 9879 | -1.473 | 0.0000 | Yes |
Table: GSEA details [plain text format]

  

Fig 2: RICHARDS\_NOWAKOWSKI\_SCIENCE\_2017\_OPC\_UPREG: Random ES distribution      
 Gene set null distribution of ES for **RICHARDS\_NOWAKOWSKI\_SCIENCE\_2017\_OPC\_UPREG**
